# Effect of cochlear implantation on vestibular function in children: A scoping review

**DOI:** 10.3389/fped.2022.949730

**Published:** 2022-09-20

**Authors:** Max Gerdsen, Cathérine Jorissen, Daphne Catharina Francisca Pustjens, Janke Roelofke Hof, Vincent Van Rompaey, Raymond Van De Berg, Josine Christine Colette Widdershoven

**Affiliations:** ^1^Department of Otolaryngology-Head and Neck Surgery, Maastricht University Medical Center, Maastricht, Netherlands; ^2^Department of Otolaryngology-Head and Neck Surgery, Antwerp University Hospital, Antwerp, Belgium; ^3^Department of Translational Neurosciences, Faculty of Medicine and Health Sciences, University of Antwerp, Antwerp, Belgium

**Keywords:** cochlear implantation, vestibular function, children, vestibular testing, video head impulse test, vestibular evoked myogenic potential, caloric test, rotatory chair test

## Abstract

**Objective:**

To provide a scoping review of the available literature for determining objectively the effect of cochlear implantation on vestibular function in children.

**Methods:**

A literature search was performed and the following criteria were applied: vestibular tests that were performed on subjects within the range of 0–18 years old before and after cochlear implantation. The papers conducted at least one of the following tests: (video) head impulse test, caloric test, cervical and ocular vestibular evoked myogenic potentials or rotatory chair test. Included papers underwent quality assessment and this was graded by risk of bias and directness of evidence.

**Results:**

Fourteen articles met the selection criteria. The included studies showed that cochlear implantation leads to a decrease in vestibular function in a proportion of the patient population. This loss of vestibular function can be permanent, but (partial) restoration over the course of months to years is possible. The pooling of data determined that the articles varied on multiple factors, such as time of testing pre- and post-operatively, age of implantation, etiologies of hearing loss, used surgical techniques, type of implants and the applied protocols to determine altered responses within vestibular tests. The overall quality of the included literature was deemed as high risk of bias and medium to low level of directness of evidence. Therefore, the data was considered not feasible for systematic analysis.

**Conclusion:**

This review implicates that vestibular function is either unaffected or shows short-term or permanent deterioration after cochlear implantation in children. However, the heterogeneity of the available literature indicates the importance of standardized testing to improve our knowledge of the effect of cochlear implantation on the vestibular function and subsequent developmental consequences for the concerned children.

## Introduction

In children, a severe-to-profound sensorineural hearing loss (SNHL) affects the quality of life and development of communicational skills (1, 2). Various studies described that hearing-impaired children show delay in motor development, presumably caused by balance disorders (3–5). Vestibular dysfunction is present in ~30–70% of children affected by SNHL (4, 6, 7). Considering that the cochlea and the vestibular system share a common embryological origin and are anatomically closely related, they are both vulnerable to similar pathologies. Hence, it is presumed that many children affected by SNHL may have concomitant hearing and vestibular loss (4, 5, 8).

Historically, the majority of vestibular research focused on adults. Research involving the vestibular function and disorders in children is more sparse (7). Firstly, this is explained by the fact that vestibular disorders in children are often not recognized. It is challenging for children to express vestibular complaints, especially for children born with vestibular dysfunction who developed unique mechanisms to compensate for this impairment. Due to these compensatory mechanisms, children with vestibular disorders generally do not present with complaints of dizziness, vertigo and/or unsteadiness, as observed in adult cases. More subtle signs of congenital or acquired vestibular dysfunction in children are a delay in both motor and cognitive development (3, 4, 9–11).

The vestibular organ is a physiologically complex system and consist of multiple components: three semicircular canals and two otolith organs, each with their own function. A wide variety of tests were invented to attempt to objectify the performance of these different components. Examples of these tests are the caloric test (video), head impulse test [(v)HIT], rotatory chair and the vestibular evoked myogenic potentials (VEMP) (12–15). A single test gives insight to part of the vestibular function, but a combination of tests addresses the overall performance of the vestibular organ more thoroughly.

A cochlear implant (CI) is a surgically implanted device for children with severe-to-profound SNHL to restore hearing and speech perception to a certain level. However, despite the significant effect of CI on hearing, the effect of cochlear implantation on vestibular function is still largely unknown (9, 16–19). Findings of earlier research are often conflicting: some studies implied that surgical damage or a decrease in vestibular function are a result of the insertion of the CI electrode (16, 20, 21), while other studies found no change in vestibular function after CI surgery (19, 22). Finally, some studies in adults demonstrated improvement of vestibular function after cochlear implantation (22, 23). Given these discrepancies in literature, no consensus is yet reached whether bilateral cochlear implantation should be performed during a single surgery, or sequentially. The advantage of sequential surgical procedures, is the fact that vestibular function can be tested after the first cochlear implantation. In case of vestibular function loss, it could be hypothesized that the pros of a bilateral cochlear implant might not outweigh the cons of a possible risk of bilateral vestibular function loss. Then, it could be decided to not perform the second implantation. However, the drawback is that two separate surgeries are needed, including risks of anesthesia etc. (24, 25).

The aim of this systematic review was to evaluate the effect of cochlear implantation on vestibular function by comparing vestibular testing before and after surgery in children.

## Methods

The literature search for this systematic review was performed following the PRISMA statement (26).

### Data sources and search strategy

The search queries (Appendix 1) were defined. They were performed in Pubmed, Embase and Cochrane. MESH-terms were only used in Pubmed, whereas Emtree terms were only used in Embase.

### Selection criteria

**Figure 1:**
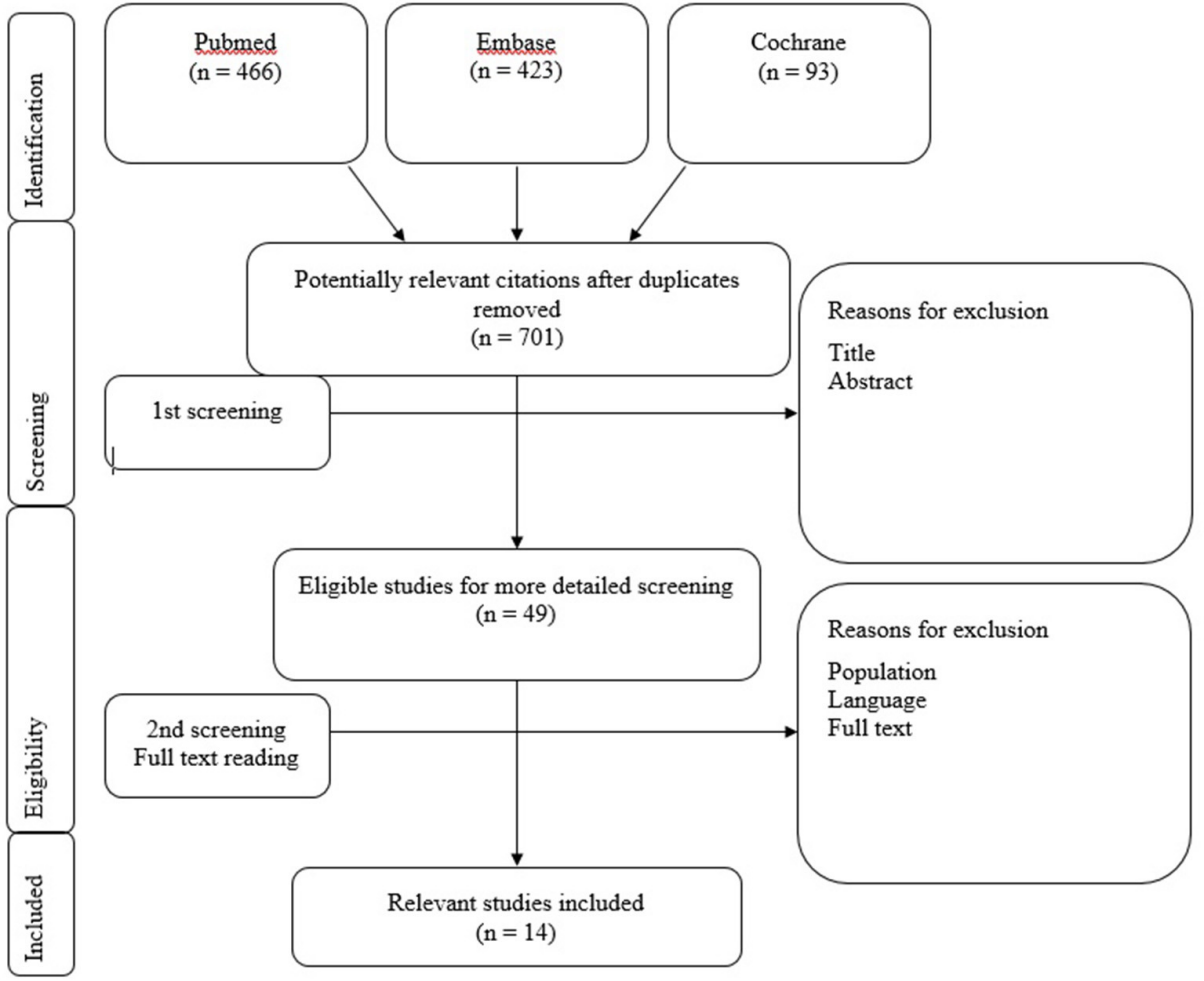
Flowchart 1: Literature selection procedure. Literature selection procedure. Included studies Jacot et al. (9), Licameli et al. (17), Dhondt et al. (27), Ajalloueyan et al. (28), Devroede et al. (29), Thierry et al. (30), Gupta and Raj (31), Guan et al. (32), Imai et al. (33), Li and Gong (34), De Kegel et al. (35), Xu et al. (36), Psillas et al. (37), and Jin et al. (38).

See Figure 1 for an overview of the selection procedure. Eligibility of the studies for the systematic review was based on the following inclusion criteria:

1. Children with sensorineural hearing loss

Participants involved children with SNHL with or without contralateral CI(s) before the respective implantation. No etiologies of the SNHL were excluded. Children were defined as having an age within the range of 0–18 years old. Articles describing a wider age range were considered when children or age groups were analyzed separately.

2. Cochlear Implantation

Studies evaluating the influence of cochlear implantation on vestibular function. The device type and surgical technique were not taken into account for determining eligibility.

3. Vestibular function tests

A minimum of one test examining the vestibular function. Tests accepted as vestibular function tests were defined as a test that evaluated the vestibulo-ocular reflex or the vestibulo-collic reflex. The accepted tests were caloric testing, rotational chair testing, (v)HIT, and cervical and ocular VEMP (cVEMP and oVEMP). Tests should be performed before and after surgery singular or multiple times at predetermined dates or periods.

4. English or Dutch

Original articles written in English or Dutch.

5. Full text available

### Selection procedure

The article screening procedure of relevant articles was performed by applying the selection criteria on title first (step 1), on abstract second (step 2) and followed by a selection on the full text (step 3). All steps in the article screening procedure were performed independently and blinded for each other's work by three researchers (C.J., D.P., and M.G.). After each screening step, a consensus meeting was held where the researchers compared their results. In case of discrepancies, titles, abstracts or full texts were screened a second time and discussed until consensus was reached. If consensus was not reached between the three researchers, a decisive opinion was provided by a fourth researcher (J.W.). All steps of the article screening procedure are presented in Flowchart 1.

### Quality assessment

The included studies were assessed on the quality of the performed tests by determining the risk of bias and directness of evidence. Risk of bias was assessed by applying the Revised Risk-of-bias tool for randomized trials as depicted by the Cochrane collaboration (39). The following criteria were considered applicable in this review: blinding, randomization process, standardization of treatment and outcome and completeness of data. The risk of bias was scored as “high” if four or more criteria were positive, “medium” if there were three positive criteria and “low” if < 3 criteria were met. The translating quality of the included articles was assessed by the directness of evidence. The grading system proposed by Atkins et al. was applied to score the directness of evidence (40). Directness of evidence was determined high if six or more, medium if four to five and low if three or less criteria were positive. Performing HIT and vHIT was defined as a single criterion.

## Results

### Study selection

The database searches in Pubmed, Embase and Cochrane resulted in a total of 982 studies. After de-duplication, a total of 701 articles were screened on title and abstract. After this screening procedure, 49 eligible articles were selected for a more detailed screening. Eventually, 14 relevant studies were included, from which data was extracted. These articles tested the vestibular function before and after cochlear implantation. The screening procedure and article selection is presented in Flowchart 1.

### Data extraction

The study characteristics are specified in Table 1. The populations of all included articles consisted of children with SNHL with or without a unilateral implanted CI. The age range varied between 12 months and 23 years old. The population sizes depicted in Table 1 addresses the individuals that underwent testing before and after implantation. This ranged from three to 89 subjects per study. Four studies used a control group consisting of children with normal hearing. All included studies performed either one or more of the predetermined tests for the data extraction.

**Table 1:** Study characteristics.

		**Cochlear implantation group**		**Control group**		**Follow-up date of vestibular testing**
**References**	**Study design**	***N***	**Age range (mean)**	***N***	**Age range (mean)**	
Ajalloueyan et al. (28)	PC	27	12–56 m (27.19 m)	-	-	6–8 w
Devroede et al. (29)	RC	24	1–13 y (6.75 y)	-	-	3 m
Dhondt et al. (27)	RCS	3	N/A	-	-	N/A
Guan et al. (32)	PC	10	6–17 y (10.0 y)	-	-	29–37 d
Gupta and Raj (31)	PCS	23	3–7 y (5.48 y)	-	-	6 w
Imai et al. (33)	PCS	4	7–13 y (9.25 y)	-	-	29–46 d
Jacot et al. (9)	CS + P	89	7 m−16.7 (51 m)	-	-	1 w−7 y
Jin et al. (38)	PC	12	2–7 y (3.8 y)	-	-	23 d−3 y
De Kegel et al. (35)	PC	19	N/A	+	N/A	6/12/18/24 m
Li and Gong (34)	PC	35	3–18 y (8.26 y)	+	4–11 y (6.3 y)	5 d/1 m/2 m
Licameli et al. (17)	CS	19	2–23 y (8 y)	-	-	4–6 w
Psillas et al. (37)	PC	10	1.5–4 y (2.85 y)	+	2–5 y (3 y)	10 d/6 m
Thierry et al. (30)	PC	12	1.2–17.2 y (4.3 y)	-	-	0.1–10.8 y
Xu et al. (36)	PC	26	3–12 y (5.52 y)	+	4–10 y (6.45 y)	1 m

PC, Prospective cohort; RC, Retrospective cohort; RCS, Retrospective case series; PCS, Prospective case series; CS + P, Cross sectional + prospective; C, Cross sectional; d, day; w, week; m, month; y, year; N/A, not available.

Follow up date: -, period of single measurement per subject; /, different timepoints of measurement per subject.

### Critical appraisal

During pooling of the data, a significant heterogeneity was identified among the respective studies on a variety of characteristics. For example, the time of testing post-operatively differed from days to years between studies (Table 1). Other aspects that varied were the age of implantation and etiology of hearing loss. Another observed discrepancy was the definition of an altered response for a specific vestibular test. Different studies used different criteria to determine whether a caloric test or VEMP was considered abnormal. Therefore, the final result per test could differ as most studies applied a dichotomous model to produce the statistical significance and subsequently the conclusion of their tests (Table 2). Due to this inconsistency between studies, it was decided to perform a scoping analysis of the available literature on the effect of cochlear implantation on vestibular function.

**Table 2:** Type of measurements and applied models to determine alterations in vestibular test results.

	**Vestibular tests**				
**References**	**Caloric test**	**Rotatory chair test**	**(video) Head impulse test**	**Cervical vestibular evoked myogenic potential**	**Ocular vestbiular evoked myogenic potential**
Ajalloueyan et al. (28)	Air stimulation (24 and 50°C); cut-off not described		Manual testing; corrective saccade (horizontal canal)	Amplitude	
Devroede et al. (29)	Irrigation (30 and 44°C); bilateral >20% asymmetry, unilateral cut-off 27%			Threshold (cut-off not specified)	
Dhondt et al. (27)	Irrigation (30 and 44°C); unilateral cut-off 18%	Gain, phase, asymmetry; patient vs. normative data^*^		Threshold (cut-off not specified)	Threshold (cut-off not specified)
Guan et al. (32)	Air stimulation (24 and 50°C); unilateral cut-off 25%		Video testing; gain (horizontal canal < 0.8, anterior and posterior canal < 0.7)	Amplitude; AR > 0.34 or no repeatable waveforms	Amplitude; AR > 0.34 or no repeatable waveforms
Gupta and Raj (31)	Air stimulation (50°C); bilateral >15% asymmetry				
Imai et al. (33)		Gain index; patient vs. normative data^*^		Amplitude; pre-op AR(%)- post-op AR (%)	Amplitude; pre-op AR(%)- post-op AR (%)
Jacot et al. (9)	Air stimulation (33 and 44°C); bilateral >15% asymmetry	Phase, amplitude; patient vs. normative data^*^		Threshold (cut-off not specified)	
Jin et al. (38)				Amplitude, latency; ratio < 0.5 vs. control	
De Kegel et al. (35)				Threshold (cut-off at 95 dB)	
Li and Gong (34)				Threshold (cut-off at 131 dB), amplitude, latency, interpeak latency	Threshold (cut-off at 131 dB), amplitude, latency, interpeak latency
Licamelli et al. (17)				Threshold (cut-off not specified), amplitude, latency	
Psillas et al. (37)				Amplitude	
Thierry et al. (30)	Irrigation (30 and 44°C); bilateral >15% asymmetry		Manual testing; corrective saccade (horizontal canal)	Threshold (cut-off at 110 dB), latency	
Xu et al. (36)				Threshold (cut-off not specified), latency, interpeak latency, amplitude	Threshold (median), latency, interpeak latency, amplitude

AR, asymmetry ratio; pre-op, pre-operatively; post-op, post-operatively.

* Normative data, values in healthy individuals obtained from a different study.

The quality of the included studies is depicted in Table 3. All but one article showed a high risk of bias for addressing the hypothesis. This was mainly contributed to level of blinding and randomization, as none of the articles met these criteria. In case of standardization of treatment, the type of implant was addressed whether it was similar for the included subjects per article. One study performed the tests prospectively with one type of implant. The remaining studies either used different implant types or did not describe which CI device (i.e., manufacturer, model, electrode, etc.) was implanted. Furthermore, it was addressed whether the studies applied a standardized follow up date for outcome measurements. Standardized outcome was defined as a predetermined follow up date or dates. This did not exclude follow up dates that differed from days to weeks. Therefore, a follow-up date that differed in days to weeks within that fixed point of time was determined as standardized, if it was addressed in the study methods. Ten of the included studies addressed the vestibular test on fixed points of time after implantation. These studies however differed in the time after implantation that the vestibular tests were performed, varying from 10 days to 3 months. For three studies it was determined that the data was partially incomplete. Overall, the included studies were determined as a high risk of bias in determining the effect of cochlear implantation on vestibular test outcomes.

**Table 3:** Quality assessment of included studies.

			**Risk of Bias (RoB)**						**Directness of Evidence (DoE)**								
											**Outcome**						
**References**	**Sample size (*n*)**	**Study design**	**Blinding**	**Treatment allocation**	**Standardization (*T*)**	**Standardization (*O*)**	**Complete data**	**Level of RoB**	**Patients**	**Therapy**	**Rotational chair**	**Caloric testing**	**HIT**	**vHIT**	**cVEMP**	**oVEMP**	**Level of DoE**
Ajalloueyan et al. (28)	27	PC	□	□	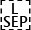	■	■	High	■	■	□	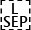	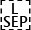	□	■	□	Moderate
Devroede et al. (29)	24	RC	□	□	■	■	■	Moderate	■	■	□	■	□	□	■	□	Moderate
Dhondt et al. (27)	3	RCS	□	□	□	□	□	High	■	■	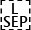	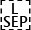	□	□	■	□	Moderate
Guan et al. (32)	10	PC	□	□	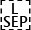	■	■	High	■	■	□	■	□	■	■	■	High
Gupta and Raj (31)	23	PCS	□	□	□	■	■	High	■	■	□	■	□	□	□	□	Low
Imai et al. (33)	4	PCS	□	□	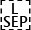	■	■	High	■	■	■	□	□	□	■	■	Moderate
Jacot et al. (9)	89	CS + P	□	□	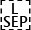	□	■	High	■	■	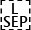	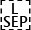	□	□	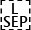	□	Low
Jin et al. (38)	12	PC	□	□	□	□	■	High	■	■	□	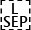	□	□	■	□	Low
De Kegel et al. (35)	19	PC	□	□	□	■	□	High	■	■	□	□	□	□	■	□	Low
Li and Gong (34)	35	PC	□	□	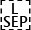	■	■	High	■	■	□	□	□	□	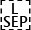	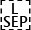	Low
Licameli et al. (17)	19	CS	□	□	□	■	■	High	■	■	□	□	□	□	■	□	Low
Psillas et al. (37)	10	PC	□	□	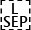	■	■	High	■	■	□	□	□	□	■	□	Low
Thierry et al. (30)	12	PC	□	□	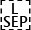	□	■	High	■	■	□	■	■	□	■	□	Moderate
Xu et al. (36)	26	PC	□	□	□	■	□	High	■	■	□	□	□	□	■	■	Low

Sample size, study population included in analysis; PC, Prospective cohort; RC, Retrospective cohort; RCS, Retrospective case series; PCS, Prospective case series; CS + P, Cross sectional + prospective; CS, Cross sectional; DoE, directness of evidence; RoB, risk of bias.

For defining the level of RoB and DoE a scale of cubes was used: a filled cube counts as 1, an empty cube as 0, and a half-filled cube as 0.5.

When the level of RoB has a score of four or more, the level was described as **low**. When the score was 3, the level was **moderate**. When the score was < 3 the level was **high**.

When the level of DoE has a score of six or seven, the level was described as **high**. When the score was four or five, the level was **moderate**. When the score was 3 or less, the level was **low**.

Assessment of Risk of Bias (RoB):
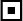

Blinding: □

- ■ = patient, researcher, and observer were blinded. - □ = no blinding of patient, researcher or observer.

Treatment allocation:

- ■ = the allocation was randomized of concealed. - □ = the allocation was not randomized or concealed.

Standardization of therapy (T):

- ■ = a uniform type of CI was implanted in all patients. - 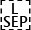 = patients received different types of CI, but the type of implant was described. - □ = the implant type was not described.

Standardization of outcome (O):

- ■ = a standardized protocol for all patients was used. - □ = no standardization or not described.

Outcome data complete:

- ■ = < 10% missing data. - □ = ≥ 10% missing data.

**Assessment of directness of evidence (DoE):**

Patients:

- ■ = all patients are < 18 years.

Therapy:

- ■ = patients underwent unilateral cochlear implantation. - □ = other.

Outcomes: rotational chair, caloric testing, (v)HIT, cVEMP, and oVEMP

- ■ = outcomes were measured pre- and postoperatively in all patients. - 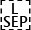 = outcomes were measured partially in the referred population. - □ = other.

Directness of evidence was determined overall to be medium to low (Table 3). One study showed a high directness of evidence. The quality of the studies was mainly influenced by the lack of more than two tests being performed per subject in a variety of studies. In all articles, all tests were specifically performed before and after unilateral implantation. It should be noted that in a majority of cases the vestibular tests were only performed on a part of the subjects post-operatively. It can be concluded that the overall directness of evidence was of suboptimal quality throughout the included papers.

### Vestibular tests

#### Caloric test

The caloric test was performed in seven of the included studies (9, 27–32). Five of the seven articles determined that the caloric test was not significantly altered after implantation. However, two articles demonstrated a significant deterioration of this test (Table 4). Of the five studies that did not find an altered response on group level, significant deteriorations were observed within the included subjects. In most papers it was a small percentage of the sample, but in case of Jacot et al. it showed that 26 of the 87 tested subjects had a decreased caloric response after implantation. This was however deemed not significant for the population size. The caloric test was performed from 6 weeks to 11 years after implantation (Table 1). Interestingly, the two studies that mentioned an altered response, performed the tests in the same period (4–6 weeks) post-operatively, whereas all but one of the aforementioned studies assessed the test with a higher variability and up to years after implantation.

**Table 4:** Post-operative effect of cochlear implantation on vestibular tests on implanted side per tested group.

	**Vestibular tests**					
**References**	**Cal**.	**Rot. chair**	**HIT**	**vHIT**	**cVEMP**	**oVEMP**
Ajalloueyan et al. (28)	x	-	x	-	x	-
Devroede et al. (29)	x	-	-	-	x	-
Dhondt et al. (27)	x	x	-	-	x	x
Guan et al. (32)	#	-	-	x	#	#
Gupta and Raj (31)	#	-	-	-	-	-
Imai et al. (33)	-	x	-	-	x	x
Jacot et al. (9)	x	x	-	-	x	-
Jin et al. (38)	-	-	-	-	x	-
De Kegel et al. (35)	-	-	-	-	x	-
Li and Gong (34)	-	-	-	-	#^*^	#
Licamelli et al. (17)	-	-	-	-	#	-
Psillas, 2014	-	-	-	-	x	-
Thierry et al. (30)	x	-	x	-	x	-
Xu et al. (36)	-	-	-	-	#	#

Cal, caloric test; Rot. Chair, rotatory chair; (v)HIT, (video) Head Impulse Test; cVEMP, cervical vestibular evoked myogenic potential; oVEMP, ocular vestibular evoked myogenic potential.

-, not performed; x, no significance; #, significant decrease of vestibular function (p < 0.05).

* Significant decrease 5 days after surgery, after 30 days restoration to normal observed.

#### Rotatory chair

Three studies reported on rotatory chair test results (9, 27, 33). All three studies concluded no significant alteration after implantation. Dhondt et al. conducted the research retrospectively with a variable post-operative date for testing (dates were not specified). The second study by Jacot et al. assessed the test in a prospective cohort. Seven subjects presented with a deteriorated response after surgery. In five cases the vestibular dysfunction was partially to completely restored within 4 days after implantation. It should be mentioned that the authors did not specify the specific test results at each time point. In case of the rotatory chair test, no overall significant alteration was described (Table 4). The last paper by Imai et al. showed that the test was altered in two cases. Whereas, one case showed a decreased gain, the other subject showed an increased gain after implantation. The tests were performed at a wide variety of dates after surgery up to 7 years (Table 1).

#### (v)HIT

Three studies assessed the effect of cochlear implantation on canal function by (v)HIT (28, 30, 32). A consistent finding was that the (v)HIT was not affected by implantation per tested group (Table 4). Again, the test was variably performed from 4 weeks up to 10 years after surgery (Table 1). The consistency of no significant altered response was however noticeable between studies and no differences were observed between the manual HIT and video HIT in post-operative results in any of the included studies. One of the more recent studies did show however that in certain individuals the test showed a decreased response. In Guan et al. 20% of the teste children showed a gain below their reference after surgery, whereas this was 0% before implantation. However, this study also described a difference in effect of implantation when vHIT was compared to other vestibular tests overall. In this case, the vHIT did not alter significantly after implantation, whereas the caloric test and VEMPs were affected post-surgery (32).

#### cVEMP

One of the most frequently performed vestibular tests in the included literature was the cVEMP (9, 17, 27–30, 32–38). This test was performed in 13 out of 14 studies. However, the overall effect of implantation on the cVEMP results showed a discrepancy throughout these articles. A noticeable difference between studies was the definition of a decreased cVEMP response. Five studies only used cVEMP amplitude and differed regarding criteria to define a decreased response. In contrast, the remaining studies used cVEMP thresholds or a combination of different cVEMP parameters (threshold, amplitude, latency and/or interpeak latency) to determine a decreased response. Most studies applied a cut-off value for the threshold to determine whether the VEMP response was present or absent. A majority of these studies did not mention the exact cut-off value. Interestingly, the three studies that specified a cut-off value, all used different values (Table 2). Four studies found a significant decrease in cVEMP response after implantation (Table 4). Interestingly, all of these studies performed the test within the first 4–6 weeks after surgery (Table 1). Li et al. described a clear restoration of cVEMP after the observed deterioration within 2 months after surgery (Table 4). Two articles did not find any effect of surgery on VEMP within the first 6 weeks (33, 37). However, these articles described that certain subjects did show deterioration of the cVEMP amplitude after surgery. The remaining studies that did not show an effect on group level, were performed in a period after 6 weeks and up to 11 years. It should be noted that these studies also demonstrated noticeable deterioration of either the cVEMP threshold or amplitude after implantation in some individuals within the population.

#### oVEMP

oVEMPs were performed in five studies (27, 32–34, 36). As described for cVEMP, these studies used different definitions to determining a decreased oVEMP response (Table 2). Overall, the majority of the papers concluded a significant decrease in oVEMP response after cochlear implantation (Table 4). It should be noted that in the study performed by Li et al., the oVEMP response was deteriorated after 2 months, but restored to a normal response when the CI was turned on in certain cases. The test was mostly performed within the first 2 months after implantation (Table 1).

## Discussion

This scoping review was performed to evaluate the effect of cochlear implantation on vestibular function in children, by comparing vestibular test results pre- and post-operatively. The included studies showed that cochlear implantation leads to a decrease in vestibular function in a proportion of the patient population. This loss of vestibular function can be permanent, but (partial) restoration over the course of months to years is possible.

It is not yet completely known why some specific patients demonstrate a loss of vestibular function after cochlear implantation. Multiple factors could contribute to this finding. Regarding vestibular function loss directly after surgery, it could be hypothesized that surgical manipulation of the inner ear might damage the vestibular system. For example, insertion of an electrode in the cochlea could create a hydraulic “shock wave” in the labyrinth, and suctioning closely to the labyrinth might result in loss of endolymphatic fluid. Furthermore, anatomical variations like an enlarged vestibular aqueduct could also account for loss of vestibular function due to per-operative endolymphatic leakage (41–43). It was previously also hypothesized that genetic predisposition, type of electrode array and some diseases (e.g., cytomegalovirus infection or bacterial meningitis) are predisposing factors for the development of vestibular function loss after implantation (44, 45). Regarding long-term effects of cochlear implantation on vestibular function, processes like tissue formation, inner ear toxicity and neo-osteogenesis could also negatively influence vestibular function (44, 46). However, overall it remains yet unclear which specific factors determine the effect of cochlear implantation on the vestibular system.

Due to the heterogeneity of the published data, it was not possible to reliably pool data. Firstly, many inter-individual differences were present, varying from different ages of implantation, etiologies (e.g., anatomic variants), used surgical techniques and type of implants. Secondly, different vestibular tests with variable protocols were used in the included studies. A limited number of studies used more than one type of vestibular examination. When comparing the outcomes of the different studies it is important to take this heterogeneity into account. For example, in the case of caloric tests, knowing exactly how the test was carried out (use of water/air conduction, exact temperatures, duration of stimulation) and at what age, is essential when comparing different outcomes. In addition, it is advised to use testing protocols for children that have also been carried out in healthy children of different ages, as normative values are not the same in children as they are in adults (47). For example, the gain of the vestibulo-ocular-reflex during head impulse testing increases during the first 6 years after birth. This could imply that children might perform better over the course of their life, while possible damage to the vestibule might go unnoticed when tested months to years after cochlear implantation. Moreover, the articles did not describe whether the tested children underwent any vestibular rehabilitation procedures, which could alter vestibular outcome measurements. Thirdly, the timing of the post-operative vestibular examinations may be a factor that hindered comparison between studies. For example, cVEMP and oVEMP responses measured with bone-conduction showed less alteration after cochlear implantation than those tested with air-conduction (48). This implies that, for example, a post-operative hematotympanum or a recently performed mastoidectomy may impact the outcomes of VEMPs and possibly also the caloric test (49). One of the included studies in this review implied that vestibular function can restore within weeks after implantation (34). When a number of vestibular tests are combined and performed at different time points after implantation, these potential confounding factors might be elucidated. Therefore, future studies should evaluate vestibular function using a comprehensive test battery pre-operatively and at fixed intervals after cochlear implantation. This test battery needs to ensure the semicircular canals and otolith system are tested with age-appropriate tools next to an evaluation of motor development. After these possible factors are explored, it might lead to identifying groups at increased risk of losing vestibular function after cochlear implantation. Identifying these groups could lead to improved decision making whether a child should undergo bilateral simultaneous implantation, sequential cochlear implant surgery, or (in the future) vestibulo-cochlear implantation (50, 51).

Vestibular test results could also be influenced by other factors. After all, studies in adults illustrated an improvement or stimulation of vestibular function after CI activation (22, 23, 52). Indeed, an increase in cVEMP responses was also found in children (53). This may be the result of the electrical current of the implant transducing from the cochlea to the vestibular system. Some of the studies in this review did not describe whether or not the results were measured with the implants switched on or off. However, it would be of interest to determine what the effect of CI activation could be on outcomes of different vestibular tests and at different timings post-surgery in future research.

Finally, it is important to note the clinical significance of an objectified loss of vestibular function. There are indications that children who underwent cochlear implantation show a delay in motor development compared to control groups, up till 2 years after surgery (35). Nevertheless, the long-term effect of implantation on the motor development of children has yet to be evaluated.

## Conclusion

Vestibular function is either unaffected or shows short-term or permanent deterioration after cochlear implantation in children. The current literature is insufficient to determine the overall effect of cochlear implantation on the vestibular system due to its heterogeneity regarding patient population, surgical procedures and vestibular testing protocols. Future studies should focus on determining specific factors which lead to loss of vestibular function due to cochlear implantation. This could eventually lead to an improved decision making process and subsequent choice of strategy in both restoring the hearing impairment and concomitantly preserving the vestibular function.

## Data availability statement

The original contributions presented in the study are included in the article/Supplementary material, further inquiries can be directed to the corresponding author.

## Author contributions

All authors listed have made a substantial, direct, and intellectual contribution to the work and approved it for publication.

## Conflict of interest

The authors declare that the research was conducted in the absence of any commercial or financial relationships that could be construed as a potential conflict of interest.

## Publisher's note

All claims expressed in this article are solely those of the authors and do not necessarily represent those of their affiliated organizations, or those of the publisher, the editors and the reviewers. Any product that may be evaluated in this article, or claim that may be made by its manufacturer, is not guaranteed or endorsed by the publisher.
