# Supplementary material for: Effect of cochlear implantation on vestibular function in children: A scoping review
Source: Front Pediatr. 2022 Sep 20;10:949730. doi: 10.3389/fped.2022.949730 (PMC9530705; doi:10.3389/fped.2022.949730)
Supplement: Supplementary file 1 [file Table_1.docx]

# Supplementary figure 1

Title: search queries

|  | MESH | EMTREE | Terms |
| --- | --- | --- | --- |
| P1 | Cochlear Implantation  Cochlear Implants | Cochlear implantion  Cochlea prosthesis | Cochlear Implantation  Cochlear Implantations  Cochlea Implantation  Cochlea implantations  Cochlear Implant  Cochlear Implants  Cochlea Implant  Cochlea Implants  CI  Cochlear prothesis  Cochlear protheses  Cochlea prothesis  Cochlea protheses  Auditory prothesis  Auditory protheses  Hearing prothesis  Hearing protheses  Cochlear prosthesis  Cochlear prostheses  Cochlea prosthesis  Cochlea prostheses  Auditory prosthesis  Auditory prostheses  Hearing prosthesis  Hearing prostheses |
| P2 | Adolescent  Infant  Child, Preschool  Child  Pediatrics | Adolescent  Infant  Child  Pediatrics | Child  Children  Infant  Infants  Adolescent  Adolescents  Paediatric  Pediatric |
| C | - |  |  |
| O | Vestibular Diseases  Labyrinth, Vestibule | Vestibular Disorder  Vestibular Labyrinth | Vestibular system  Vestibular labyrinth  Vestibular disorder  Vestibular imbalance  Vestibular function  Vestibular loss  Vestibular damage  Vestibular manifestations  Vestibular syndrome  Vestibular disease  Vestibular hypofunction  Vestibular failure  Vestibular impairment  Vestibuloccular reflex  Vestibular stimulation  Vestibular |

**Search Pubmed**

(("Cochlear Implantation"[Mesh] OR "cochlear implants"[Mesh] OR "Cochlear Implantation"[All Fields] OR "Cochlear Implantations"[All Fields] OR "Cochlea Implantation"[All Fields] OR "Cochlea implantations"[All Fields] OR "Cochlear Implant"[All Fields] OR "Cochlear Implants"[All Fields] OR "Cochlea Implant"[All Fields] OR "Cochlea Implants"[All Fields] OR "CI"[All Fields] OR "Cochlear prothesis"[All Fields] OR "Cochlear protheses"[All Fields] OR "Cochlea prothesis"[All Fields] OR "Cochlea protheses"[All Fields] OR "Auditory prothesis"[All Fields] OR "Auditory protheses"[All Fields] OR "Hearing prothesis"[All Fields] OR "Hearing protheses"[All Fields] OR "Cochlear prosthesis"[All Fields] OR "Cochlear prostheses"[All Fields] OR "Cochlea prosthesis"[All Fields] OR "Cochlea prostheses"[All Fields] OR "Auditory prosthesis"[All Fields] OR "Auditory prostheses"[All Fields] OR "Hearing prosthesis"[All Fields] OR "Hearing prostheses"[All Fields]) AND ("Adolescent"[Mesh] OR "Infant"[Mesh] OR "Child, Preschool"[Mesh] OR "Child"[Mesh] OR "Pediatrics"[Mesh] OR "Child"[All Fields] OR "Children"[All Fields] OR "Infant"[All Fields] OR "Infants"[All Fields] OR "Adolescent"[All Fields] OR "Adolescents"[All Fields] OR "Paediatric"[All Fields] OR "Pediatric"[All Fields]) AND ("Vestibular Diseases"[Mesh] OR "Vestibule, Labyrinth"[Mesh] OR "Vestibular system"[All Fields] OR "Vestibular labyrinth"[All Fields] OR "Vestibular disorder"[All Fields] OR "Vestibular imbalance"[All Fields] OR "Vestibular function"[All Fields] OR "Vestibular dysfunction"[All Fields] OR "Vestibular loss"[All Fields] OR "Vestibular damage"[All Fields] OR "Vestibular manifestations"[All Fields] OR "Vestibular syndrome"[All Fields] OR "Vestibular disease"[All Fields] OR "Vestibular hypofunction"[All Fields] OR "Vestibular failure"[All Fields]
